# Intratumoral immunotherapy prior to cancer surgery, a promising therapeutic approach

**DOI:** 10.3389/fimmu.2025.1545000

**Published:** 2025-06-18

**Authors:** Kevine Silihe Kamga, Steven Fiering

**Affiliations:** Department of Microbiology and Immunology, Dartmouth Geisel School of Medicine, Hanover, NH, United States

**Keywords:** cancer, immunotherapy, neoadjuvant, abscopal, intratumoral, *in situ* vaccination

## Abstract

Cancer immunotherapy has made astonishing progress in the last 10–15 years, and the rate of progress is accelerating. However, only 20 to 40% of patients benefit from this therapy with most immunotherapy applied post discovery of metastatic disease when therapeutic impact is more difficult to achieve. The first line of treatment for many patients following diagnosis is surgery. Neoadjuvant immunotherapy, i.e. administration of immune therapy prior to surgery, has the potential to improve overall survival rates. Many patients without detectable metastases are diagnosed with a high risk of future metastasis and could benefit from effective neoadjuvant immunotherapy. An ideal neoadjuvant immune therapy will stimulate immune response against the identified tumor as well as undetected metastasis and be safe with minimal adverse events. In addition, the antitumor immune response it generates should not be blocked by subsequent surgery and should not delay the normal timeline of surgery. Finally, it should be relatively inexpensive. These features describe intratumoral immunotherapy (ITIT), a therapeutic approach that directly administers immune stimulatory agents or treatments into the tumor. By delivering the therapy directly into the tumor, it enhances local drug concentration while minimizing nonspecific immune activation and adverse events associated with systemic immunotherapy. ITIT can generate effective local immune response against tumor antigens, which expands the pool of tumor-recognizing effector T cells. ITIT induces and activates tumor specific T cell within days after the treatment, so surgery is not delayed. Tumor-recognizing effector T cells generated locally attack cancer both locally and systemically, targeting metastasis through the “abscopal effect”. Neoadjuvant ITIT options are extensive and expanding and need research into optimal options to combine and associated dosing and timing. With the needed effort, neoadjuvant ITIT will develop into a safe, rapid and effective addition to current cancer therapies.

## 1 Introduction

Immunotherapy is an established therapy for many cancers. It acts by modulating the immune system primarily through the stimulation of immune effector cells that recognize the tumors. Commonly used immunotherapies systemically administer antibodies to block immune checkpoint molecules on predominantly T cells, PD-1/PD-L1 and CTLA-4 (checkpoint blockade therapy) (1). Although checkpoint blockade therapies (CBT) have remarkable efficacy for some patients with many tumor types, most patients have minimal response and for many patients CBT is limited by auto-immune reactions (2), cytokine storm (3) and other adverse events. CBT currently has high financial cost (4) and is not likely going to be available to most patients in low resource countries. As with many cancer therapies, relapses often occur in those who do respond, due to the development of resistance (1). Clearly there is a need to expand the options for cancer immunotherapy to help more patients, with greater safety and less cost.

The strong clinical benefits observed with patients receiving immunotherapy after surgery has increased interest and usage of cancer immunotherapies before surgery (5). This interest is supported by preclinical data from mouse models showing that administering neoadjuvant intratumoral immunotherapy (ITIT) increased both local and systemic antitumor immunity (6–9).

Neoadjuvant therapy, treatment prior to surgical resection, is generally used to reduce tumor size in order to reduce the extent of surgery required, sparing healthy tissue and thereby decrease the morbidity of the surgical procedure (10). Clinical studies validate advantages of neoadjuvant therapies for cancer, and established applied modalities include chemotherapy, radiotherapy, hormone therapy or systemic immunotherapy (11).The concept put forth here of neoadjuvant ITIT is not focused on treated tumor reduction, although it could be the outcome. The concept is to use ITIT to 1) disrupt the local tumor-generated immune suppression, 2) enable effective antigen presentation of tumor antigens in the draining lymph nodes, 3) generate increased systemic antitumor immunity, particularly from T cells that can eliminate nascent metastases. This takes advantage of the understanding that every cancer therapy generally is more effective when tumor burdens are low. To optimize this strategy, neoadjuvant ITIT should: be safe with minimal adverse events, be rapid and have its impact without delaying surgery from its normal timeline since delaying surgery could enable metastasis from the primary tumor (12, 13), be deliverable, and be reasonably inexpensive so usable even in low resource situations.

Intratumoral immunotherapy administers immune stimulatory agents/treatments directly into one or more recognized tumors. While this generally mediates shrinkage of the treated tumor, that is not the primary purpose of neoadjuvant ITIT. Successful neoadjuvant ITIT should reduce metastasis and relapse primarily by expanding tumor-recognizing effector T cells which increases systemic anti-tumor immunity and impacts tumors at distant sites (the “abscopal effect”) (14). Additionally, it can induce immunological memory that protects patients against reactivation of dormant tumor cells (5). While it takes weeks or months to fully manifest the effects of successful ITIT, the local immune changes it initiates occur only days after the local treatment and are minimally inhibited by subsequent surgical resection (6, 9, 15) so would not need to delay tumor resection.

Different classes of molecules are used in ITIT preclinically and in clinical trials that elicit a potential antitumor immune response. They range from microorganisms (bacteria like *Salmonella typhimurium and Clostridium novyi*); viruses (oncolytic viruses or viral vectors for gene therapy); small and macromolecules (TLR 7/8 agonists); proteins (cytokines, checkpoint antibodies); nucleotide-based gene products (IL-12 plasmids, mRNA); cells (autologous/allogeneic DCs, CAR-T or xenogeneic tissue cells) (16). While various intratumoral immunotherapy approaches are applicable in the neoadjuvant setting, adoptive cell therapies could generally not be done as neoadjuvant ITIT without significant delay in surgery and therefore are not within the focus of this review.

**Figure 1:**
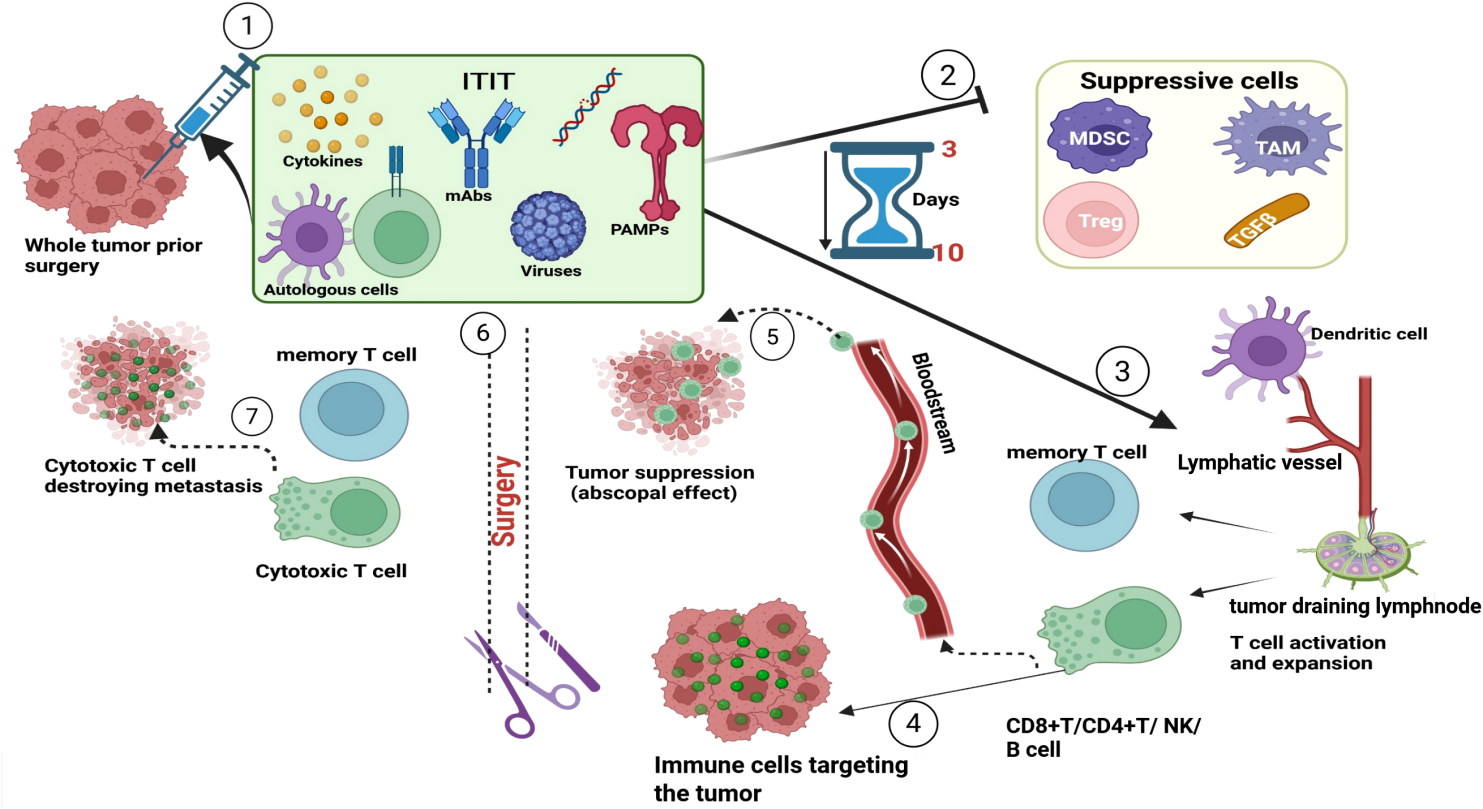
Representation of neoadjuvant intratumoral immunotherapy. Before surgery, the tumor is locally treated with immunostimulatory agents (mAb, PRR, TLR, cytokines, viruses, cells). These agents in the TME will suppress immunosuppressive cells and molecules and stimulate a robust immune response within 3 to 10 days of injection. Once the immune response is activated immune cells (CD8+T, CD4+T, NK, B cell) will target antigens present on the tumor and others will migrate through the bloodstream and recognize and attack metastases (abscopal effect). Once surgery is conducted, the circulating immune cells will continue to destroy tumors carrying the same antigens that were present in the treated tumor. Once tumors are eliminated, some cells will differentiate into memory T and B cells that respond to any future antigen exposure. mAbs, monoclonal antibodies; PAMPs, pathogen associated molecular pattern; MDSc, Myeloid derived suppressor cells; TAM, Tumor associated macrophages; TGFβ, tumor growth factor beta.

Of 130 completed neoadjuvant immunotherapy trials prior to 2020 (5), 24 used intratumoral immunotherapy. Since 2020, 18 more trials have used neoadjuvant ITIT (clinicaltrial.gov). We believe there is significant value in neoadjuvant ITIT and expect increased preclinical and clinical research to establish its clinical value alone or in combination with other immunotherapy strategies. see **Figure 1** for graphical representation of neoadjuvant intratumoral immunotherapy.

## 2 Intratumoral immunotherapy

### 2.1 History of ITIT

Dr. William Coley published the first ITIT studies in the 1890s when he injected live or dead bacteria or bacterial extracts into tumors to stimulate an immune response (17, 18). Although poorly understood then, it constituted the first well-documented attempt to leverage the immune system by a localized immune activation but had sporadic further study, in part due to the emergence of radiotherapy (19) and later, chemotherapy.

Almost one hundred year later, clinical efforts using ITIT again intensified. In 1977 Bacillus Calmette-Guerin (BCG), an attenuated mycobacteria was adopted as adjuvant treatment for nonmuscle invasive bladder cancer (20, 21). BCG instillation into the bladder post-resection remains the standard of care to suppress metastasis for nonmuscle-invasive bladder cancer. Cytokine-based ITIT using IL-2 and GM-CSF was utilized in clinical trials for the treatment of melanoma and bladder cancer (22, 23) The development of oncolytic viruses marked a step forward in ITIT with the 2015 Food and Drug Administration (FDA) approval of T-VEC, the first FDA approved oncolytic virus, which is intratumorally delivered (24). There have been further clinical trials of ITIT including gene therapies (IL-12 plasmid, IL-23 mRNA, CD40 mRNA, OX40L mRNA) (25–27); cell therapy [Dendritic Cell (DC)] (28, 29); toll-like receptor (TLR) agonists (TLR7/8/9) (30, 31), oncolytic viruses (T-VEC, HF 10, Orien X010) (32–34). More recently, STING pathway agonists have been studied for ITIT (35) and presently, ITIT is being studied in combination with systemic CBT [(36); NCT03842943], radiotherapy to enhance immune response and abscopal effect [(37); NCT01347034].

### 2.2 Characteristics of ITIT

**Figure 2:**
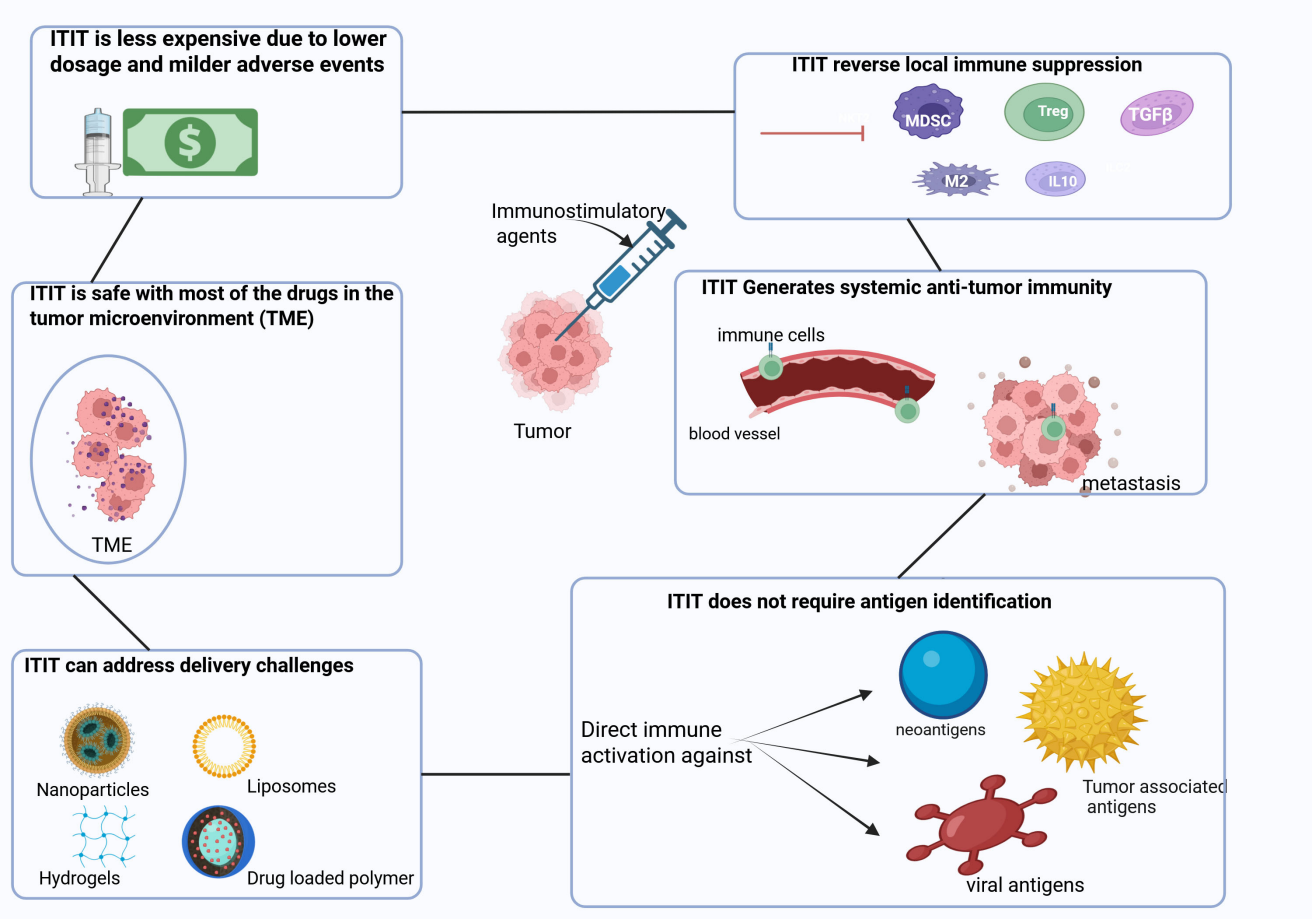
Characteristics of Intratumoral immunotherapy. ITIT has several advantages such as reversing the local immune suppression induced by immunosuppressive cells and molecules (Tregs, MDSCs, TAM, TFGβ, IL-10). Moreover, ITIT can induce a robust signal which enables the migration of immune cells through the bloodstream to target metastasis. ITIT is able to generate broad antitumor immunity against neo-antigens, tumor-associated antigens as well as viral antigens. With the use of nanoparticles, hydrogels, liposomes and drug loaded polymer, ITIT could overcome some delivery challenges ensuring a slow and sustained release of immunotherapeutics. With immunotherapeutics injected locally, ITIT is safe since most of the drug is in the tumor microenvironment avoiding systemic non-specific immune activation. With immunotherapeutics administered locally, a lower dose could be used and thus no or milder adverse events occur. Tregs: regulatory T cells, MDSCs: myeloid derived suppressor cells, TAM: tumor associated macrophages, TGFβ: transforming growth factor beta.

**Figure 2** summarizes basic characteristics of intratumoral immunotherapy.

#### 2.2.1 ITIT can reverse tumor-mediated local immune suppression

Tumor-mediated immune suppression remains the main barrier to immunotherapy since tumors create a highly immunosuppressive local environment that helps them evade immune surveillance (38). This suppression is mediated in variable and somewhat patient-specific ways including recruitment of immune suppressive leukocytes like regulatory T cells (Tregs) and myeloid-derived suppressor cells (MDSCs), expression of suppressive molecules like PD-L1, secretion of immunosuppressive cytokines like TGF β and IL-10, hypoxia, poor vascularization and other metabolic challenges for the immune system, and high extracellular matrix density that restricts infiltration of immune cells (39, 40). ITIT is designed to overcome these immunosuppressive barriers and stimulate a robust antitumor immune response. This is accomplished in part by overcoming physical and drug delivery barriers by directly applying immune stimulating treatments to recognized tumors (41).

ITIT responses vary between approaches and tumors/tumor models, but there are some general expectations. ITIT modifies the immunosuppressive TME through various pathways such as: reprogramming or reducing numbers of immunosuppressive cells including Tregs (42) TAM (43) and MDSC (44). Moreover, ITIT can modulate the TME by disrupting stromal barriers (41), modifying vasculature (45) and thus enhancing immune cell infiltration and activation (44). Representative preclinical studies include (42) where the intratumoral administration of a RIG-I agonist, led to an increase in the Teff/T reg ratio. Intratumoral administration of L-pampo, a TLR2/3 agonist led to M1 macrophage polarization and T cell activation in MC38 model (43). In Shirota et al. (46) intratumoral administration of TLR agonist reduced the immunosuppressive activity of MDSC and caused their differentiation into macrophages with direct tumoricidal capability. Intratumoral administration of poly I:C decreased myeloid-derived suppressor cells (MDSC) and attenuated their immunosuppressive activity (47) Clinical trials demonstrate the ability of ITIT to modify immunosuppressive TME or cells (48, 49). In these studies, the local administration of T-VEC led to a significant decrease in Treg/Teff ratio, and myeloid-derived suppressive cells (MDSC) in stage III and IV melanoma patients. The options for ITIT are extensive and rapidly expanding, and different tumor types and patients will likely respond better to some options than others, but we assume that any tumor can have its immune suppression modulated to immune stimulation with appropriate treatment.

#### 2.2.2 ITIT can generate systemic anti-tumor immunity

While local immune stimulation can be mediated by effective ITIT, the most important goal of neoadjuvant ITIT is expanded systemic antitumor immunity. The term “abscopal effect”, (literally “out of the scope”), was first used in 1953 when regression of distant metastases was seen in patients receiving local radiation therapy (50). Although not well understood, it was speculated that the effect utilized immunological mechanisms (51, 52). Subsequently, ITIT with nonradiation modalities was observed to trigger systemic immune responses capable of shrinking distant metastases and validating the immune basis of the abscopal effect (53). ITIT induces a pro-inflammatory environment that supports local effector immune cell recruitment and function and can generate systemic anti-tumor immune activation (54, 55). Effective ITIT generally recruits and activates antigen presenting cells (APC) such as dendritic cells (DC 1 and DC2), macrophages and B cells. These APC then migrate to lymph nodes to prime immune cells against tumor antigens (56). Activated immune cells, notably cytotoxic CD8+ T cells as well as helper CD4+ T cells (57), NK (26), and B cells (58) expand and may migrate through the body to attack metastases (59). The abscopal effect can be improved by systemically administering checkpoint inhibitors with ITIT thus allowing T cells to function more effectively, both locally and systemically (60).

#### 2.2.3 ITIT is rapid since it does not require antigen identification

A strength of ITIT is its ability to be repeatedly applied and mediate local immune stimulation in the average 10–20-day window between pathologic diagnosis and surgery. Most efforts to generate therapeutic cancer vaccines start by sequencing tumors and identifying prospective neoantigens based on mutations the tumor carries (61, 62). ITIT avoids the delay and expense of identifying target tumor antigens before stimulating antitumor immune responses (14). Intratumoral immunotherapy is also called “*in situ* vaccination”. Vaccines of any type include antigens which are the target of the vaccine, and immune stimulating reagents (immune adjuvants), which stimulate an immune response to the antigens. Tumor neoantigens are highly variable between patients, particularly since the vast majority of neoantigens are due to bystander mutations that have little or no benefit for the tumor. ITIT delivers the immune stimulation agent directly into the tumor and can stimulate antitumor immune responses against any antigen in the tumor, whether neoantigen, tumor-associated antigen or viral antigen in virally caused tumors.

ITIT can turn the tumor itself into a vaccine (*in situ* vaccination) by disrupting the tumor-mediated immune suppression of antitumor immunity (63). The tumor’s antigens are then more effectively presented to the immune system which initiates or expands an immune response (59). An additional task for antigen-carrying cancer vaccines is matching the patients HLA alleles to the new potentially presentable neoantigen peptides to select peptides with the highest probably of presentation in a specific patient. For ITIT neither antigen nor human leukocytes antigens (HLA) information is required because neither must be manipulated; ITIT uses whatever antigens exist and the existing HLA of the patient, reducing complexity and cost, and increasing speed of delivery and response. Thus, in a resectable tumor, ITIT can be performed without delaying surgery, an important advantage.

#### 2.2.4 Engineered ITIT delivery systems can address delivery challenges

Effective intratumoral injections require precise delivery of the drug into the target lesion and may also depend on the distribution of the drug throughout the tumor (64). Direct injection is feasible almost anywhere in the body by using image guidance. However, tumors not located near the skin are a challenge to inject and the treatment risk tissue damage. Most neoadjuvant ITIT with injectable reagents has utilized multiple injections with a few days or a week between treatments. ITIT could be delivered with controlled release delivery mechanisms such as slow-releasing and retentive polymeric drug vehicles (64). Nanoparticles, liposomes, hydrogels and other advanced biomaterials could deliver ITIT agents ensuring a slow and sustained release of the therapy and avoiding multiple injections (3, 65–69). This is illustrated by studies using hydrogel for the extended release of STING agonists, a nanofluidic drug-eluting seed was used to release agonist monoclonal antibodies OX40 and CD40 intratumorally, and an elastin-like polypeptide was used for the sustained release of CpG oligodeoxynucleotide immunostimulant (70–72). These studies show successful synergistic effects with distant metastasis reduction as well as greater inhibitory effects on the local tumor.

#### 2.2.5 ITIT is safe

Due to the localized administration of reagents and associated lowered overall reagent dose, ITIT is safer than systemically applied Immunotherapy. Its administration is direct for superficial, visible cutaneous, subcutaneous or cervical mucosal sites and palpable lymph nodes (16). ITIT ensures that immunotherapeutics are at high local concentration within the tumor microenvironment (TME) but low systemic concentration minimizing nonspecific general immune activation (59). This reduces immune attack of healthy tissues and associated serious adverse events, and mild local adverse events have been reported in clinical trials (NCT03259425; NCT00289016**;** 33). Adverse events of ITIT that do occur are due to the local inflammation that is the inherent goal of ITIT. The local nature of potential adverse events also makes them more clinically manageable. Limited or absent adverse events due to local neoadjuvant administration of immunotherapy allows the scheduled surgery to occur without interruption due to adverse events and supports patients’ quality of life.

#### 2.2.6 ITIT is less expensive

The high cost of current immunotherapy cancer treatments reduces access to these treatments (73). This financial constraint makes current systemic CBT-based immunotherapy unavailable to most patients in low resource countries (74, 75). The need for supportive care due to side effects of systemic immunotherapy further increases the cost of systemic CBT (76). While all clinically applied reagents are expensive, cost is reduced when the dosing is lower and when the reagents are not personalized for each patient. ITIT allows for lower doses, reducing costs while maintaining therapeutic efficacy (5, 14). Neoadjuvant ITIT as discussed here includes rapid application to complete multiple treatments without disrupting the normal surgical timeline, which precludes personalized reagents that depend on either the patient’s own cells or inclusion of tumor neoantigens specific for the patient, thus keeping expense lower than other immune therapy options.

With immunotherapy reagents concentrated in the tumor, smaller doses of the neoadjuvant reduce the overall treatment dosage, further limiting expense (5, 14). These lower drug dosages translate into reduced costs for both the medication itself and the associated medical care. Antitumor immunity is increased systemically following neoadjuvant ITIT which reduces the need for postoperative systemic treatments (chemotherapy, immunotherapy) that are often given after surgery. This is demonstrated in a clinical trial (77) where only 11% of patients received an adjuvant treatment after Talimogene laherparepvec (T-VEC) neoadjuvant ITIT in comparison to 29% of patients in the surgery only group (NCT02211131).

### 2.3 Experimental options combined with ITIT

ITIT development requires evaluation of many options experimentally and clinically. There is a large and expanding range of targets and approaches that could be used for ITIT and combinations exponentially increase the number of options. Finally dosing and timing are other variables that must be studied to optimize ITIT. Researchers are currently testing many new ways to induce localized immune activation that elicits a systemic response. A variety of oncolytic viruses (OV) are commonly studied as ITIT and generally express immune-stimulating factors (cytokines, chemokines, immune checkpoint inhibitors) (78–81). Since the review is focused on neoadjuvant ITIT that could be done rapidly without delaying surgery, we are not including adoptive cell therapies. We have expanded examples of ITIT combined with other immune therapy approaches that can be rapidly delivered with standardized reagents. Examples include OV designed to deliver therapeutic proteins or genes encoding immunotherapy reagents like anti-CTLA-4 or anti-PD-L1, directly into the TME (82, 83). OV in combination with CBA (84,NCT02509507, NCT02978625). non-viral bacteria-derived targeted oncolytic agent (VAX014) (85). STING and TLR agonists are also studied preclinically and in clinical trials since they stimulate immune responses by activating innate immune cells and generating cytokines/chemokines (72, 86) and in combination with CBA (35, 87). Cytokines such as IL-12 (88), IL-2 variants (89–91) are evaluated for ITIT. Chemotherapeutics and immunotherapeutics have been combined intratumorally (92–95). In addition, ITIT is being combined with local radiation therapy to enhance immunogenic cell death and increase the release of tumor antigens (44, 96–99). As seen in preclinical studies, the local administration of checkpoint blockade antibodies (CBA) can increase the local activation and infiltration of T-cells against tumor cells (100, 101). The targeted goal of the different combinations is aimed at maximizing immune activation thus improving therapeutic outcomes.

## 3 Potential therapeutic impact of ITIT as neoadjuvant treatment

### 3.1 Neoadjuvant ITIT targets metastasis

Metastasis is the primary source of morbidity and mortality in most solid cancers (102) and undetected micrometastases are the main cause of post-surgical relapse (103). Traditional oncologic thinking is that any treatment meant to suppress metastatic disease must be systemically applied and local treatments do not impact already established untreated metastases. This thinking was applied by both immunologists and drug companies and the widely used checkpoint blockade antibodies are systemically administered. However, when systemic neoadjuvant treatments (like immunotherapy or chemotherapy) are used to target micrometastatic disease, the risk of side effects and expense increases (104) and if metastases do not actually exist, systemic neoadjuvant therapies expose patients to unnecessary side effects.

ITIT, whether neoadjuvant or not, impacts metastatic disease by 1) reversing local tumor-mediated immune suppression of the treated tumor; 2) stimulating effective antitumor response against the treated tumor; 3) generating increased numbers of tumor recognizing lymphocytes that circulate, encounter metastases and attack them using what are generally found to be standard cell-mediated cytotoxicity mechanisms. The reversal of immune suppression enables antigen presentation cell (APC) maturation and antigen capture by antigen-presenting cells which migrate to the draining lymph nodes to activate T cells (105). Once activated, these tumor antigen-specific T-cells expand and circulate and can encounter metastases and attack them when they are very small. This enhances the infiltration of immune cells into distant tumors which amplifies the immune response beyond the treated ITIT sites (16). In general, abscopal effects require CD8 T cells and priming of CD8 by cross-presenting conventional DC1 (106, 107). While each immune response will differ, generally an immune response initiated at a specific anatomic site generates a systemic immune response against the relevant antigens. This is illustrated by vaccines against respiratory pathogens administered into the arm. While that is straightforward and obvious for immunoglobulins, it is also mainly true for cell mediated responses. Neoadjuvant intratumoral immunotherapy (ITIT) is a promising approach to cancer treatment since it addresses not only the primary tumor but also potential micrometastases. Prior to surgical resection, the intact tumor has a large amount of any recognizable tumor antigen, so ITIT at that point can exploit this mass of tumor antigens to enhance T cell diversity and priming (7)). This enables an increase in the breadth and durability of tumor-specific effector T-cells which can circulate and target metastatic diseases and initiate development of immunological memory before the tumor is removed by surgery (108). This is demonstrated in a clinical trial which administered T-VEC plus surgery (arm 1) in comparison to surgery alone (arm 2), in stage III-IV melanoma patients, a pathological complete response (pCR) of 17.1% was observed in arm 1 in comparison to 2.1% in arm 2 (77). This trial was 12 weeks of T-VEC, so it is not the type of rapid ITIT discussed here that is done without disrupting normal surgical timeline, however, it does illustrate the potential to use ITIT to generate an abscopal effect.

### 3.2 Neoadjuvant ITIT can be effective without delaying surgery

Minimizing the time lapse between pathological diagnosis and surgical resection is important for preventing metastatic disease if it has not already occurred (109). The expectation is that surgery should be done as quickly as possible so that the primary tumor is removed and does not generate metastases in the time between diagnosis and resection. When neoadjuvant radiation or chemotherapy is used to reduce the tumor to enable surgery, there is still a clear expectation that surgery should be delayed as little as possible (110). Depending on the type of cancer, the present recommended maximal time of neoadjuvant treatment to reduce a tumor before surgery is 4–6 weeks (111) for non-small cell lung cancer or 6–9 weeks (112) for melanoma. Delay of surgery due to neoadjuvant tumor-reducing treatment can increase frequency of metastatic disease, as seen in (113, 114) affecting overall survival.

Exposure of the tumor microenvironment to high doses of therapeutic agents through ITIT ensures a rapid modulation of the TME, transforming an immunosuppressive environment into an immune landscape with increased pro-inflammatory cytokines such as IFN-γ, TNF-α, IL-12, increased activated effector cells and reduced numbers of suppressive cells. This reversal and its impact occur within days, and this activates APCs after which they carry their antigen load to the draining lymph nodes where they present antigen to T cells (105, 115). The immune stimulation activates existing and generates new immune effector cells like CD8+ cytotoxic T cells and NK cells (116) within a short time frame.

Once the draining lymph nodes are activated against the tumor, the immune response in the tumor can be stopped by surgery without disrupting the development of systemic anti-tumor immunity. The window to conduct neoadjuvant immunotherapy without delaying surgery is roughly 2 weeks. Following ITIT, dividing CD8+ Tcells cells (Ki67^+^) peak in the tumor at 7-10 days (117, 118). In fact, while it takes weeks or months to fully manifest the effects of successful ITIT, the immune changes it initiates occur within a few days after the local treatment and are minimally inhibited by subsequent surgical resection. Preclinical studies (6, 9, 15, 119, 120) have shown that despite surgical tumor resection a few days after ITIT, significant systemic immune population changes could be observed and had beneficial effect against untreated tumors.

Overall, these data show that neoadjuvant ITIT can induce rapid TME modulation and immune changes that can generate systemic anti-tumor immunity without delaying the normal surgical timeline and that the systemic immunity can continue to develop after the tumor removal.

### 3.3 Neoadjuvant ITIT induces an immunological memory that protects against recurrence

Immune memory is part of the goal for neoadjuvant ITIT. Immunological memory can reduce the risk of recurrence due to quiescent tumor cells by maintaining long-term immune surveillance against the tumor.

Overall, multiple preclinical studies establish that a three to ten-day interval following ITIT prior to resection was sufficient to inhibit recurrence and metastasis (6, 42, 43, 121, 122). In (9), mice that receive CpG and aOX40 and the 4 day window was eliminated, a much higher proportion of mice had local recurrence (5/10). In comparison, the group that received a single injection of CpG and aOX40 followed by a 4-day window prior to resection had local recurrence in only 2/10 mice.

Mice who received first ITIT then surgery were challenged with the same tumor cells and rejected or had significantly slower growth of these tumors when compared to mice treated with surgery alone, indicating increased systemic immunity, a precursor for immunological memory. In contrast, challenge with unrelated tumor cells following ITIT and surgery was not affected by treatment, which demonstrates immune specificity (5). Since surgery is rarely able to be accomplished in less than 10 days following pathologic diagnosis, the apparent week or less needed for ITIT to generate antitumor immunity can be done without impacting the surgical schedule.

## 4 Preclinical evaluation of neoadjuvant ITIT reagents, approaches, combinations, dosing and timelines

Identifying the best options for ITIT and how that varies by cancer types is a challenge that must be met to enable expanded clinical usage of neoadjuvant ITIT. There is a vast array of intratumoral immunotherapeutic options, including microbes, small molecules, proteins, nucleic acid-based products, and cells that could be tested individually or in combination (16, 123). New options emerge often and the task of evaluation individually and in combinations using primarily mouse models continues (5, 123). Compounds intratumorally tested as neoadjuvant therapy include: immune stimulating receptor agonists (TLRs, STING and others) (124) (9, 125), protein (CD 40) (125); antibodies as CBT or to stimulate cells (OX 40) (9); RNA (BO-112) (15); noninfectious viral-like particles [Cowpea Mosaic virus (CPMV)] (120); oncolytic viruses (126), cytokines (IL-12, FLT3L) (125, 127, 128) and dendritic cells (128). Physical treatments like radiation (129), heat (6, 130) or cold can also contribute to ITIT. These studies support the expected benefit of local administration of immune stimulation, to inhibit metastasis and improve survival in a neoadjuvant setting.

Although reagent combinations are not yet very widely tested for ITIT, it is safe to assume that depending on the tumor and patient they will provide better efficacy than single ITIT reagents. Timing and dosing of ITIT combinations adds further complexity to identifying the best clinical options. While there are significant challenges, combinatorial treatment is the standard for most oncology efforts. Preclinical combinatorial studies will be used to delineate useful combinations and associated parameters, which must then be further optimized and ultimately validated in clinical trials. The process is in motion and new ITIT neoadjuvant combinatorial approaches are likely to be clinically tested and approved at increasing rates.

## 5 Clinical studies of ITIT in the neoadjuvant setting

In 2020, (5) reported 24 clinical trials that had used intratumoral agents as neoadjuvant treatment. Since that publication, eighteen more clinical trials have been registered at the clinicaltrial.gov website using intratumoral immunotherapy prior to surgery (**Table 1**).

**Table 1:** Ongoing trials utilizing neoadjuvant intratumoral immunotherapy.

ID TRIAL	Intratumoral agent	Combination	Tumor type	Status
NCT04526730	Tavokinogene Telseplasmid(ITIT IL-12 plasmid electroporation)	Nivolumab	Melanoma	Active, not Recruiting
NCT06472661	polyICLC (polyinosinic-polycytidylic acid	Focused ultrasound ablation (FUSA)	Melanoma	Recruiting
NCT05980598	TransCon TLR7/8 Agonist	Pembrolizumab TransCon IL-2 β/γ	Head and neck cancer	Recruiting
NCT04599062	talimogene laherparepvec (T-vec)	Radiation	Soft tissue sarcoma	Active, not Recruiting
NCT04427306	T-VEC		Melanoma	Suspended
NCT03300544	T-VEC	Capecitabine, Fluorouracil, Leucovorin, Oxaliplatin, radiation therapy	Rectal cancer	Terminated
NCT03972046	T-VEC	Dabrafenib, Trametinib	Melanoma	Withdrawn
NCT02779855	T-VEC	Paclitaxel, doxorubicin, cyclophosphamide	Breast cancer (TNBC)	Active, not recruiting
NCT02211131	T-VEC		Melanoma	Completed
NCT06347705	Anti-CD40 agonist antibody		Prostate cancer	Recruiting
NCT06736379	Virus Replicon Particle- encapsulated saRNA encoding IL-12	anti-PD-1	Head and Neck Cancer Squamous Cell Carcinomas (HCSCC)	Not yet recruiting
NCT06358573	INT230-6 (cisplatin, vinblastine, shao)		TNBC	recruiting
NCT04316091	Superparamagnetic Iron Oxide Nanoparticles (SPIONs)	Conventional chemotherapy	Osteosarcoma	unknown
NCT06014086	PH-762		squamous cell carcinoma, melanoma, or Merkel cell carcinomas of the skin	recruiting
NCT01329809	JX-594 (Thymidine Kinase-Deactivated Vaccinia Virus Plus GM-CSF)		Metastatic Colorectal Carcinoma	terminated
NCT06660810	T-VEC	radiation	soft tissue sarcomas	Not yet recruiting
NCT02723838	REOLYSIN^®^	Gemcitabine and Cisplatin	muscle-invasive bladder cancer	withdrawn
NCT03842943	T-VEC	Pembrolizumab	melanoma	recruiting

Of the 18 neoadjuvant ITIT clinical trials recorded since 2020, 4 could be categorized as either suspended/withdrawn or with an unknown status. 2 are not yet recruiting, 6 are currently recruiting, 3 are active and 3 have been completed.

The immunostimulatory agents given IT were mainly oncolytic viruses (9); gene therapy (5); PAMPs and analogs (3); and chemotherapy (1). These ITIT treatments were mainly given in combination with CBA (4); chemotherapy (4); radiation (2) and targeted therapy (1).

Overall, no serious adverse events were observed (37, 77, 131) with the local administration of immunostimulatory agents. The ITIT at the neoadjuvant setting was able to inhibit recurrence in most of the patients (37, 77, 131) and a 25% reduction in the risk of disease recurrence is estimated in patients who received ITIT plus surgery compared to surgery alone (77). Additionally, 2-year progression-free frequency was higher in patients receiving ITIT in the neoadjuvant setting compared with surgery alone (77, 131).

## 6 Concluding remarks/summary

Neoadjuvant ITIT can stimulate systemic antitumor immune responses to reduce the risk of metastasis from either established metastases or from future relapse due to dormant tumor cells. Optimal neoadjuvant immunotherapy should have proven local and systemic therapeutic value, be rapid enough to not delay surgery, have minimal side effects and not be too expensive. With such options available, cancer pathologic diagnoses that identify high potential for metastasis could stimulate neoadjuvant immunotherapy to attempt to eliminate metastases that are not yet detectable. Considerable academic and commercial research is currently focused on demonstrating the best intratumoral immunotherapy alone or in combination with other immune therapies. The effort is young but progressing rapidly as the basic idea of neoadjuvant ITIT gains acceptance. Other important goals are determining the best tumor type for a given ITIT approach and identifying biomarkers that would guide specific reagent usage. Thus, there is a need for more preclinical studies to identify the best ITIT therapy options and understand their local and systemic mechanisms of action.

Feasibility of direct delivery of ITIT reagents varies due to tumor locations and is part of the challenge that must be addressed to make neoadjuvant ITIT a standard of care. For easily accessible tumors such as skin melanoma, breast cancer, head and neck cancer, neoadjuvant ITIT delivery is less difficult. More challenging locations for injections are still almost certainly injectable by surgeons or interventional radiologists using advanced imaging technology. Fewer procedures are always preferable if it provides equal outcomes. Stable formulations suitable for localized delivery that increase retention and enable slow release could be used for deep-seated tumors that are more challenging to inject. Studies aimed at identifying the optimal dosage as well as the frequency of administration must be understood to optimize impact.

The immune microenvironment phenotype plays a crucial role in the outcome of the therapy, just as it does for any cancer immunotherapy. Tumors heavily infiltrated by CD8 T cells, are likely to be more responsive than “cold tumors” with low T cell infiltration and more immune suppressive cells. This heterogeneity in response extends to “immune desert tumors” characterized by a lack of immune cells within the tumor making it difficult to activate pre-existent immune machinery. Tumors manifest different immune suppressive mechanisms and combinations of such mechanisms. The major immune suppressive cells are generally some combination of T reg cells and immune suppressive myeloid cells. These different types of cells also manifest different suppressive mechanisms. The immune suppressive phenotype of tumors varies and optimal ITIT treatment will likely require sufficient understanding to match the ITIT approach to the immune suppressive mechanisms. The fundamental concept remains the same, the tumor is immune suppressive, to varying extents and with variable mechanisms driving that suppression, and ITIT can directly oppose that immune suppression by delivering powerful immune stimulatory signals directly to tumors.

Scientific study of intratumoral immunotherapy is over 100 years old but the application of modern understanding and approaches has generated many new strategies and understanding of molecular mechanisms involved. With currently available insights and reagents and further investment to expand options and mechanistic understanding, neoadjuvant ITIT promises to evolve into a widely used therapeutic modality for cancer therapy.
